# Willingness to accept malaria vaccine among caregivers of under-5 children in Southwest Ethiopia: a community based cross-sectional study

**DOI:** 10.1186/s12936-022-04164-z

**Published:** 2022-05-12

**Authors:** Getachew Asmare

**Affiliations:** Department of Reproductive Health and Nutrition, School of Public Health, College of Health Science and Medicine, Wolaita Sodo University, Wolaita Sodo, Ethiopia

**Keywords:** Willingness, Acceptance, Malaria, Vaccine, Under five, Caregiver

## Abstract

**Background:**

Malaria is widespread in Ethiopia and has been a major cause of illness and death in that country. Therefore, Ethiopia has been exerting enormous efforts towards eliminating malaria by 2030. In the context of comprehensive malaria control, the malaria vaccine is used for the prevention of *Plasmodium falciparum* malaria in children living in regions with moderate- to-high malaria transmission.

**Methods:**

A community-based cross-sectional study was conducted among caregivers of children under the age of five throughout the months of September 2021. A structured interviewer-administered questionnaire was designed for data collection, and binary logistic regression analysis was used. The final result of the association was determined based on an adjusted odds ratio (AOR) at a 95% confidence interval (CI) level, and p < 0.05 indicated statistical significance.

**Results:**

A total of 406 caregivers of children under the age of 5 were interviewed. Overall, 131 (32.3%) respondents were willing to vaccinate their children. Marital status (AOR = 1.243; 95% CI 1.021–3.897), knowledge (AOR = 3.120; 95% CI 1.689–5.027), and previous experience with childhood vaccination (AOR = 2.673; 95% CI 1.759–4.101) were found to be significantly associated with willingness to accept a malaria vaccine for their children, at p < 0.05.

**Conclusions and recommendations:**

The willingness to accept a malaria vaccine for children among caregivers of children under the age of five was low in the study area. Thus, health education and communication are crucial for alleviating poor knowledge about malaria vaccines.

**Supplementary Information:**

The online version contains supplementary material available at 10.1186/s12936-022-04164-z.

## Background

Malaria is a vector-borne disease transmitted through the bites of Anopheles mosquitoes. A number of species of the genus *Plasmodium* cause malarial infections in humans, but most cases are caused by either *Plasmodium falciparum* or *Plasmodium vivax* [1]. Malaria continues to be a major global health problem, with over 40% of the world’s population being exposed to varying degrees of malaria risk in some 100 countries [2]. In Africa, there were 228 million malaria cases and 602,000 deaths in 2020. This continent accounted for about 95% of cases and 96% of deaths globally; 80% of all deaths in this region are among children aged under 5 years [3].

The spread of malaria is severe in Ethiopia and has been a major cause of illness and death [4]. It is estimated that 75% of the country’s land is malarious with about 68% to70% of the total population living in at- risk areas [4–6]. *Plasmodium falciparum* and *P. vivax* are the most dominant malaria parasites in Ethiopia, accounting for 60% and 40% of malaria cases, respectively [7]. According to the 2005 Ethiopian Demographic Health Survey, malaria was the primary cause of health problems, accounting for 17% of outpatient visits, 15% of hospital admissions and 29% of in-patient deaths [8]. The Ethiopian Malaria Indicator Survey [9] estimates, the prevalence of malaria to be 1.3%. It is also well evidenced that Ethiopia, one of the sub-Saharan country in Africa, shares 6% of the burden [10]. Recent public media sources have revealed that Ethiopia has been exerting enormous efforts to eliminate malaria by 2030. The country is working to eliminate the disease in 565-selected malaria-prone *woreda* (the third level of administrative division of Ethiopia after zones and regional states) [11].

The World Health Organization (WHO) recommends indoor residual spraying (IRS), insecticide-treated nets, larval control, preventative chemotherapy and early diagnosis and treatment to prevent and control the disease [12, 13]. Increasing parasite resistance to anti-malarial drugs, poor income, poor IRS compliance, inadequate access/unfair distribution of health care, insufficient health service infrastructures and high population movement influences the effectiveness of malaria prevention and control in the country [14].

In response to the drawbacks of current malaria control strategies, the United Nations Development Programme, World Bank and WHO-Tropical Diseases Research programme, in collaboration with pharmaceutical industry experts have explored other novel approaches to malaria control, one of which is the use of vaccinations [15]. Vaccination is a public health strategy that is very effective in disease control and has successfully eradicated some infectious diseases, such as smallpox and is currently very promising in eradicating poliomyelitis [16].

A malaria vaccine is under evaluation in Ghana, Kenya, and Malawi for use as a complementary control tool [13]. RTS, S/AS01 (RTS, S) (Mosquirix™) is the world’s first licensed and protein-based recombinant malaria vaccine to protect against *P. falciparum* [17, 18].

In the context of comprehensive malaria control, the WHO recommends that the RTS,S/AS01 malaria vaccine should be used for the prevention of *P. falciparum* malaria in children living in regions with moderate-to-high transmission rates. The RTS,S/AS01 malaria vaccine should be provided on a schedule of four doses for children from 5 months of age to reduce malaria disease and burden. To date, more than 2.3 million doses of the vaccine, which has a favourable safety profile, have been administered in three African countries [19].

The provision of four doses of the RTS,S/AS01 vaccine, when complemented with other preventive and control packages to children above 5 months of age, given at 6, 7.5, 9, and 18 months after the third dose [20], causes a significant reduction of malaria morbidity and mortality [21].

The first and third doses of the vaccine align with 6-month vitamin A supplementation and 9-month first dose of the measles vaccine, respectively. The fourth dose is given 15–18 months after the third dose. This new vaccine would require two additional immunization contacts to align with the current expanded programme of immunization in Ethiopia [22].

Understanding communities’ willingness to receive a malaria vaccine and the main factors influencing their willingness will help in the development and implementation of effective means of promoting malaria vaccine uptake and facilitating the recent malaria elimination programme in the country. However, no prior studies have been conducted in the study area, Ethiopia in particular. Therefore, the current study has investigated the willingness of caregivers of children under the age of five to accept the malaria vaccine for their child in Southwest Ethiopia.

## Methods

### Study design, period, and setting

A community-based cross-sectional study was conducted among caregivers of children under the age of five in Woliata Sodo town, southwest Ethiopia throughout the month of September 2021. The town is located 380 km south of Addis Ababa, the capital city of Ethiopia. Administratively the town has 11 *kebeles* (the smallest administrative unit in Ethiopia). According to the 2021 report of the town health office, 244,817 people and an estimated 25,537 under-five children lived in the town and were served by one public referral hospital, one private hospital, three health centres, and other private health facilities [23].

### Source and study population

All caregivers of children under the age of five in the town were source population and those who had chances to participate in the selected kebeles were the study population.

### Inclusion and exclusion criteria

All permanent resident caregivers of children under the age of five in the selected household during data collection were eligible to participate. However, caregivers of children under the age of five who were seriously ill and unable to respond were excluded.

### Sample size and sampling procedure

The required sample size was calculated by using single population proportion formula with an assumption of 95% (CI), 5% margin of error, 50% proportion of willingness towards the malaria vaccine among caregivers of children under the age of five (because there is no study done so far). The sample size was found to be 384. After adding a 10% non-response rate, the final sample size was 422. The town has eleven kebeles. Six kebeles (Damota, Kera, Fana,Wadu,Hibret and Gido) were selected randomly. Households with children of caregivers under the age of five were listed out from family folder of health extension workers (HEW) of randomly selected kebeles and the samples were allocated proportionally to each selected kebeles. Finally, study respondents were selected by simple random sampling technique.

### Data collection tools, measurements and quality control procedures

A structured interviewer-administered questionnaire was used for data collection. The questionnaire was adopted and modified from different works of literatures [24–29]. Before distributing, its validity was checked by a panel of experts in the field, and ascertained its content validity. Pretest was also done among 5% of the sample size in Selam kebele. It was then amended according to the comments raised by the experts and pretest result. Reliability analysis was done and Cronbach’s Alpha had been larger than 0.7, indicating good internal consistency in the responses. It was prepared in the English language, then translated to a local language, and translated back to English to maintain its consistency.

The questionnaire had three parts. The first part comprised questions regarding personal socio-demographic information, the second part consisted of health-related information, the third part covered the knowledge and willingness related variables. Three diploma nurse data collectors, and one-degree nurse supervisors were assigned. The training was given for data collectors and supervisors for 2 days on the way of administering and gathering the questionnaire. The completeness of the questionnaire was also checked before data entry (Additional files  1 and 2).

Comprehensive knowledge about the malaria vaccine was computed from summing up all relevant six knowledge-related questions about the malaria vaccine. The correct answer for each item was scored “1” and the incorrect answer was scored “0.” Accordingly, respondents who scored greater than or equal to the mean value of the sum of knowledge assessment questions were thought as having good knowledge, and respondents who answered less than the mean value of the sum of knowledge assessment questions were thought as having poor knowledge.

Willingness to accept malaria vaccine was assessed by one item “are you willing to vaccinate your child for the future when the vaccine is available?“ Those who responded yes were scored “1” and no was scored “0.”

### Data processing and analysis

The collected data were coded and entered into Epi-Data version 3.1 and then was exported to SPSS version 25.0 for analysis. Descriptive statistics were done by computing summary statistics like frequency, mean, percentages, and standard deviations, and the results were presented in tables. Binary logistic regression was done to assess the crude relationship between the independent variables and willingness to accept malaria vaccine. All variables having a P ≤ of 0.2 were considered as a candidate for multivariable logistic regression to control for possible confounding effects. Multivariable logistic regression was applied to see the independent effect of each variable on the outcome variable. The final result of the association was determined based on AOR at a 95% CI level and p < 0.05 indicted statistical significance.

## Results

### Socio-demographic characteristics of caregivers of under-5 children

A total of 406 caregivers of children under the age of five were interviewed making a response rate of 96.2%. From the total caregivers who participated in this study, 193 (47.5%) were between the age groups of 31–40 years. The mean age of respondents was (32.7 ± 6.6). The majority, 249 (61.3%) of respondents were orthodox Christians. Three hundred and twenty-six (80.3%) of the caregivers were married. About 329 (81%) of caregivers of children under the age of five were females. The occupational status of 141(34.7%) of caregivers were merchants and 132(32.5%) were government employees. One hundred and seventy-four (42.9%) of respondents had a primary level of education. Three hundred and five (75.1%) of the caregivers/parents had less than four children. Concerning average monthly family income of caregivers, 228(56.2%) of respondents earn between 1000 and 5000 ETB monthly. Three hundred and three (74.6%) of caregivers were biological parents to their child (Table 1).

**Table 1:** Socio-demographic characteristics of caregivers of under-5 children in southwest Ethiopia in 2021 (N = 406)

Variables	Category	Frequency	Percent
Age	< 30	175	43.1
	31–40	193	47.5
	41–50	30	7.4
	51–60	8	2
Sex	Male	77	19
	Female	329	81
Marital status	Married	326	80.3
	Unmarried	80	19.7
Education	Unable to read and write	33	8.1
	Read and write	64	15.8
	Primary education(1–8)	174	42.9
	Secondary education and above	135	33.3
Average Monthly Income	< 1000 ETB	112	27.6
	1000–5000 ETB	228	56.2
	5000–10,000 ETB	48	11.8
	≥ 10,000 ETB	18	4.4
Occupation	Government employee	132	32.5
	Private employee	57	14
	Merchant	141	34.7
	Housewife	59	14.5
	Others	17	4.2
Religion	Orthodox Christian	249	61.3
	Muslim	41	10.1
	Protestant	94	23.2
	Others	22	5.4
Family size	< 4	305	75.1
	≥ 4	101	24.9
Number of under-five children	1	299	73.6
	≥ 2	107	26.4
Relationship caregiver to the child	Biological parents	303	74.6
	Grandparents	43	10.6
	Relatives	49	12.1
	Others	11	2.7

### Health-related characteristics of respondents

The majority 351 (86.5%) of respondents had previous experience with childhood vaccination. Seventy-nine (19.5%) of caregivers and sixty-six (16.3%) of children were suffered from malaria within the last year (Table 2).

**Table 2:** Health-related characteristics of caregivers of under-5 children in southwest Ethiopia in 2021 (N = 406)

Variables	Category	Frequency	Percent
Previous experience with childhood vaccination	Yes	351	86.5
	No	55	13.5
Caregivers suffered from malaria within the last year	Yes	79	19.5
	No	327	80.5
Child suffered from malaria within the last year	Yes	66	16.3
	No	340	83.7

### Knowledge about malaria vaccine

Seventy-three (18%) of respondents ever heard about the malaria vaccine. The majority of respondents got information from Facebook, news from national TV/radio, and health care providers with a percentage of 53.3%, 15.1%, and 15.1% respectively. Overall, 100 (24.6%) of respondents had good knowledge about malaria vaccine respondents (Table 3).

**Table 3:** knowledge about malaria vaccine among caregivers of under-5 children in southwest Ethiopia in 2021 (N = 406)

Variables	Category	Frequency	Percent
Ever heard about malaria vaccine	Yes	73	18
	No	333	82
source of information	from news from national TV/radio	11	15.1
	from government agencies	4	5.5
	Social media (Facebook, telegram	39	53.3
	discussion amongst friends and families	8	11
	health care providers	11	15.1
Knowledge about vaccine	Good	100	24.6
	Poor	306	75.4

### Willingness to accept malaria vaccine

One hundred and thirty-one (32.3%) of respondents were willing to vaccinate their child for the future when the vaccine was available. The main reason for not being willing to vaccinate their child is because they think that if not given orally it may paralyze the child (33.5%), maybe expensive (26.5%) and their husbands will not support vaccination (15.3%) (Table 4).

**Table 4:** Willingness to accept malaria vaccine among caregivers of under-5 children in southwest Ethiopia in 2021 (N = 406)

Variables	Category	Frequency	Percent
Willingness to accept malaria vaccine	Yes	131	32.3
	No	275	67.7
If no, reason	My husband does not want to vaccinate our child	42	15.3
	The vaccine may be expensive	73	26.5
	If not given orally, may paralyze the child	92	33.5
	No money to treat adverse effect of vaccine	16	5.8
	Immunization against malaria reduces the fertility rate of children when they grow up	7	2.5
	Culture forbids child vaccination	25	9.1
	Religion forbids child vaccination	20	7.3

### Factors associated with willingness to accept malaria vaccine

All sociodemographic, health-related characteristics and knowledge about the vaccine were entered into bivariable logistic regression. Marital status, knowledge, and previous experience of childhood vaccination were found to be significantly associated with willingness to accept malaria vaccine at p < 0.05 in multiple logistic regression.

Caregivers of children under the age of five who were married were 1.2 times more likely to have willingness to vaccinate their child compared to unmarried caregivers (AOR = 1.243; 95% CI  1.021–3.897). Caregivers of the child under the age of five who had good knowledge about the malaria vaccine were three times more likely to willingness to vaccinate their child compared to those who had poor knowledge about the vaccine (AOR = 3.120; 95% CI 1.689–5.027). Caregivers of the child under the age of five who had previous experience with childhood vaccination were 2.7 times more likely to have willingness to vaccinate their child compared to those who had no previous experience (AOR = 2.673; 95% CI 1.759–4.101) (Table 5).

**Table 5:** Factors associated with willingness to accept malaria vaccine among caregivers of under-5 children in southwest Ethiopia in 2021 (N = 406)

Variables	Willingness		COR (95% CI)	AOR (95 % CI)	p-value
	Yes	No			
Sex					
Male	22	55	1.239 (0.718–2.136)	1.354 (0.865–3.102)	0.320
Female	109	220	1	1	
Marital status					
Married	106	220	0.943 (0.557–1.597)	**1.243 (1.021–3.897)**	0.021*
Unmarried	25	55	1	**1**	
Knowledge					
Good	27	73	1.392 (1.044–2.297)	**3.120 (1.689–5.027)**	0.000*
Poor	104	202	1	1	
Family size					
< 4	97	208	1.088 (0.675–1.755)	1.187(0.964–3.083)	0.621
≥ 4	34	67	1	1	
Previous experience about childhood vaccination					
Yes	111	240	1.236 (1.082–2.237)	**2.673 (1.759–4.101)**	0.002*
No	20	35	1		
Caregivers attacked by malaria within the last year					
Yes	21	58	1.400 (1.008–2.425)	1.600(0.876–3.303)	0.411
No	110	217	1	1	

* Statistically significant at p < 0.05

## Discussion

Vaccination is not the only, but the best solution to controlling infectious diseases [30]. However, while most people vaccinate according to the recommended schedule, the success is being challenged by individuals and groups who choose to delay or refuse vaccines [31]. Vaccine hesitancy is believed to be responsible for decreasing vaccine coverage and an increased risk of vaccine-preventable disease outbreaks and epidemics [32]. Ethiopia has been exerting enormous efforts to eliminate malaria by 2030. The country is working to eliminate the disease in 565 selected malaria-prone woreda [11]. The innovation of the malaria vaccine will have a significant effect on the malaria elimination strategy. Understanding communities’ willingness to receive a malaria vaccine and the main factors influencing their willingness towards it will help to develop and implement effective means of promoting malaria vaccine uptake and to facilitate the recent malaria elimination programme in the country.

In this study, 131 (32.3%) of respondents were willing to vaccinate their child for malaria in the future when the vaccine was available. The finding of this study was lower than a study done in Abuja Nigeria (98%), Calabar Nigeria (53%), Ibadan Nigeria (87%), Tanzania (94.5%), Kenya (88%), and the rural community of Nigeria (91.6%) [24–29]. he difference may be due to differences in sociodemographic features of study respondents, time of the study, sample size, and study design.

The malaria vaccine is an innovative health intervention in Ethiopia [33]. PATH MVI stated that experience has shown that the development of an innovative health intervention does not necessarily mean that it will be adopted, delivered, accepted, and used immediately in a way that will make a significant impact on people’s health. There are several, interrelated technical, individual, political, financial, and social issues that influence the adoption and implementation of new health interventions. Late attention to these issues is likely to result in a delayed policy decision regarding a health technology or in a decision being taken without enough information to support it and facilitate its use [16].

The main causes of hesitancy to vaccinate their child towards malaria vaccine were; the vaccine may paralyzed the child if not given orally, expensiveness of the vaccine, refusal of partners. The finding was consistent with a study done in Ibadan, Nigeria [26]. Respondents in this study requested that the vaccine be given free, suggesting that they might not be able to afford the costs or that they are not willing to pay for the vaccine. This could have been borne out of the fact that vaccines for child immunization in the country are currently free. However, findings of research conducted in Ethiopia [22], 60.6% of caregivers of under-five children were willing to pay for the childhood malaria vaccine for US$ 23.11 per full dose.

In this study, caregivers’ marital status, knowledge, and previous experience with childhood vaccination were found to be significantly associated with willingness to accept malaria vaccine at p < 0.05.

Caregivers of the child under the age of five who were married were 1.2 times more likely to have willingness to vaccinate their child compared to unmarried caregivers. The finding was consistent with a study done in Nigeria, India, Kenya and Japan [34–37]. This may be due to single mothers lack time for child healthcare [37]. To solve this problem, improvement in the working conditions among single mothers is needed. Furthermore, for example, simplification of vaccination systems, institutionalization of paid leave for child healthcare, and vaccination programmes at the mother’s workplace can facilitate access to child vaccination for single mothers who are busy with both home and work.

Caregivers of the child under the age of five who had good knowledge about the malaria vaccine were three times more likely to have willingness to vaccinate their child compared to those who had poor knowledge about the vaccine. The finding was consistent with a study done in Calabar Nigeria, Tanzania, and Kenya [25, 27, and 36]. This is because the effectiveness of vaccines relies on both clinical efficacy and a community’s knowledge [38]. During vaccine promotion, lack of community support due to poor knowledge and perceptions resulted in poor community uptake while others reject vaccines [39]. Therefore, health education and communication from government sources are very crucial methods to alleviate the poor knowledge about malaria vaccine.

Caregivers of the child under the age of five who had previous experience with childhood vaccination were 2.7 times more likely to have willingness to vaccinate their child compared to those who had no previous experience. The finding was consistent with a study done in Ibadan Nigeria, Ethiopia, and Vietnam [22, 26, and 40]. The possible reasons might be that caregivers who had previous experience might have adequate information about the advantage and side effect of the vaccine to prevent vaccine preventable diseases. Therefore, understanding which factors are consistently associated with the decision to vaccinate one’s child is important to identify messages, which should be targeted by public health communications about routine child vaccinations.

## Conclusions and recommendations

Willingness to accept malaria vaccine among caregivers of the child under the age of five was low in the study area. Marital status, knowledge, and previous experience of childhood vaccination were found to be significantly associated with willingness to accept the malaria vaccine. Understanding which factors are consistently associated with the decision to vaccinate one’s child is important to identify messages, which should be targeted by public health communications about routine child vaccinations. Health education and communication from government sources are very crucial methods to alleviate the poor knowledge about malaria vaccine. Since the vaccine is not yet available, the concerned bodies shall ensure the availability of the malaria vaccine.

## Supplementary Information

**Additional file 1: **English language version questionnaire.

**Additional file 2: **Amharic language version questionnaire.

## Data Availability

The datasets used and/or analysed during the present study are available from the corresponding author on reasonable request.

## Abbreviations

AOR: Adjusted odds ratio
CI: Confidence interval
ETB: Ethiopian Birr
IRS: Indoor residual spraying
PATH MVI: People Assisting The Homeless Malaria Vaccine Initiative
