# Supplementary material for: Willingness to accept malaria vaccine among caregivers of under-5 children in Southwest Ethiopia: a community based cross-sectional study
Source: Malar J. 2022 May 12;21:146. doi: 10.1186/s12936-022-04164-z (PMC9097094; doi:10.1186/s12936-022-04164-z)
Supplement: Supplementary file 1 — Additional file 1: English language version questionnaire. [file 12936_2022_4164_MOESM1_ESM.docx]

# English language version questionnaire

**Woliata Sodo University College of Health Science and medicine**

**Department of Reproductive Health and Nutrition**

**Kebele--------------------------**

**Code of respondent------------------------------**

# Information Sheet

Questionnaires on assessment of willingness to accept malaria vaccine among caregivers of under five children in Southwest Ethiopia.

How are you? My name is (Name of the data collector) ____________________. We are conducting health research on assessment of willingness to accept malaria vaccine among caregivers of under five children.

This is beneficial to identify areas of improvement in malaria vaccine acceptance among caregivers of under five children and highlighting the need for corrective actions. By doing this we will provide sufficient information for policy makers, clinicians so that they could make informed decision. I would like to inform you that you are chosen to be interviewed. Before we go to the interview, I will request you to listen carefully to what I am going to read to you about the purpose and general condition of the study and tell me whether you agree or disagree to participate in this study.

**Consent Form**

The purpose of this study is to identify areas of improvement in willingness to accept malaria vaccine among caregivers of under five children and highlighting the need for corrective actions. The study will be conducted through interviews. The interview will only take about 15-20 minutes of your time. At the end, it is hoped that the information you give us could help to improve malaria vaccine acceptance among caregivers of under five children and hence decrease under five mortality due to malaria. The interview involves private life questions. I would like to assure you that this privacy should strictly be kept confidential. A code number will identify every participant and no name will be used. The interview is voluntary and there will not be any incentives. You have the right to respond or not respond to the all or some questions. You can also stop the interview in between if you are not interested. Your participation or nonparticipation, or refusal to respond to the questions will have no effect now or in the future on services that you or any member of your family may receive from service providers.

If you have any question you may contact: Getachew Asmare Mobile phone: *+251960808193, Email gasmare35@gmail.com*

Are you willing to participate in this study?

1. Yes 2. No

Thank you!!

If the study subject agrees to participate in the study, start the interview.

Interviewer signature certifying that informed consent has been given verbally by the respondent.

Name of the interviewer________________ Signature _____________Date ______________

Name of supervisor __________________ Signature_______________ Date_____________

# Sections of the questionnaire

General instruction: ask the following questions then circle their answer on response column if choice question or write the response on blank space if open ended question.

**Section one. Socio-demographic predictor variables**

| Questions | Response | Remark |
| --- | --- | --- |
| Age | ---------------- |  |
| Sex | 1. Male 2. Female |  |
| Marital status | 1. Married 2. Unmarried |  |
| Religion | 1. Orthodox 2. Muslim 3. Protestant 4. Others |  |
| Education | 1. Unable to read and write 2. Able to read and write but no formal education 3. Primary education(grade 1-8) 4. Secondary education (grade 9-12) and above |  |
| Occupation | 1. Government employee 2. Private employee 3. Merchant 4. Housewife 5. Others |  |
| Monthly income in ETB | -------------------------- |  |
| Family size | ------------------------ |  |
| Number of under five children | --------------------- |  |
| Relationship of the caregiver with the child | 1. Biological parent 2. Grand parent 3. Relatives 4. Others |  |

**Section two. Health related variables**

| Question | Response | Remark |
| --- | --- | --- |
| Do you have previous experience about childhood vaccination? | 1. Yes 2. No |  |
| Have you suffered from malaria in last year? | 1. Yes 2. No |  |
| Did your child suffered from malaria in last year? | 1. Yes 2. No |  |

**Section three. Knowledge and willingness related variables**

| Question | Response | Remark |
| --- | --- | --- |
| Have you ever heard about malaria vaccine? | 1. Yes 2. No |  |
| If yes, what was source of information? | 1. from news from national TV/radio 2. from government agencies 3. Social media (Facebook, telegram 4. discussion amongst friends and families 5. health care providers |  |
| Do you know about advantage malaria vaccine? | 1. Yes 2. No |  |
| For which age group the vaccine will be given? | 1. For under five years old child 2. For five years and above child 3. I do not know |  |
| Do you know about the effectiveness of the malaria vaccine? | 1. Yes 2. No |  |
| How much full dose of vaccine given to protect from malaria? | 1. Two doses 2. Four doses 3. I do not know |  |
| Does the vaccine has side effect? | 1. Yes 2. No |  |
| Is it very dangerous for health using malaria vaccine overdoses? | 1. Yes 2. No |  |
| Are you willing to vaccinate your child for the future when the vaccine available? | 1. Yes 2. No |  |
| If no, why? | -------------------------------- |  |

Thank you‼!
