# Supplementary material for: Willingness to accept malaria vaccine among caregivers of under-5 children in Southwest Ethiopia: a community based cross-sectional study
Source: Malar J. 2022 May 12;21:146. doi: 10.1186/s12936-022-04164-z (PMC9097094; doi:10.1186/s12936-022-04164-z)
Supplement: Supplementary file 2 — Additional file 2: Amharic language version questionnaire. [file 12936_2022_4164_MOESM2_ESM.pdf]

ወላይታ ሶዶ ዩኒቨርስቲ ጤና ሳይንስና ህክምና ኮሌጅ

ስነ-ተዋልዶ እና ስነ ምግብ ትምህርት ክፍል

ቀበሌ-------------------

የመጠይቁ መለያ ኮድ------------------

<u>አጠቃላይ መረጃ መስጠት</u>

ጤና ይስጥልኝ! ስሜ (መረጃ ሰብሳቢዉ ስም) ______________________ይህ መጠይቅ በደቡብ ምዕራብ ኢትዮጵ የሚኖሩ ከአምስተ አመት በታች ህፃን ያላቸዉ ተንከባካቢወችን የዉባ ክትባት ለልጆቻቸዉ ለማስከተብ ያላቸዉን ፈቃደኝነት እና ተዛማጅ ምክኒያቶች ዳሰሳ የሚያደርግ መጠይቅ ነዉ፡፡ .እኔ የጥናቱ መረጃ ሰብሳቢ ነኝ ፡፡ ይህን በማጥናት ለፖሊሲ አዉጪዎች እና ለለህክምና ባለሙያዎች በቂ የሆነ መረጃ በመስጠት በመረጃ ላይ የተመሰረተ ዉሳኔ እንዲወስኑ ያግዛል፡፡አርስዎን ለቃለ መጠይቅ መርጠንዎታል፡፡ወደ ቃለ መጠይቁ ከመግባታችን በፊት ግን ስለ ጥናቱ አላማ እና አጠቃላይ ሁኔታ የማነብልዎትን እንዲያዳምጡኝና በጥናቱ ለመሳተፍ ፈቃደኛ መሆንዎንና አለመሆዎን አንድታረጋግጡልኝ እጠይቅዎታለሁ፡፡

<u>ፈቃደኛነትን መጠየቂያ ቅፅ</u>

የዚህ ጥናት አላማ ከአምስተ አመት በታች ህፃን ያላቸዉ ተንከባካቢወችን የዉባ ክትባት ለልጆቻቸዉ ለማስከተብ ያላቸዉን ፈቃደኝነት እና ተዛማጅ ጉዳዮች የሚያጠናና መሻሻል አና መስተካከል ያለባቸዉን ክፍተቶች የሚጠቁም ጥናት ነዉ፡፡ጥናቱ የሚሰበሰበው በቃለ መጠይቅ ይሆናል፡፡ ቃለ መጠይቁ የሚወስደው ጊዜ ከ15 እስከ 20 ደቂቃ ይሆናል፡፡ ቃለመጠይቁ የግል ህይወት ጥያቄዎችን ያካትታል፡፡ ይህ የሚሰጡት መረጃ በሚስጥር እንደሚጠበቅ ላረጋግጥልዎት እወዳለሁ፡፡ እያንዳንዱ ተሳታፊ በሚስጥር ቁጥር ይለያል ስም አይጠቀስም፡፡ ቃለመጠይቁ በፈቃደኝነት ላይ የተመሰረተ ሲሆን ምንም አይነት ድጎማ የለውም ፡፡ ጥያቄዎቹን ሙሉ በሙሉም ሆነ በከፊል የመመለስ ሙብት አለዎት፡ እንዲሁም ካልተመቸዎት በመሀል ቃለመጠይቁን ማቆም ይችላሉ፡፡ የእርስዎ መሳተፍ ወይም አለመሳተፍ አሁንም ሆነ ወደፊት እርሶም ሆነ ቤተሰብዎ በሚያገኙት አገልግሎት ላይ የሚያመጣው ጉዳት አይኖርም፡፡ በጥናቱ ዙሪያ ማንኛውም ጥያቄ ካለዎት ከዚህ በታች የተጠቀሱትን ሰዎች በሚፈልጉት ጊዜ ማነጋገር ይችላሉ፡፡ ጌታቸዉ አስማረ ኢሜል፤ gasmare35@gmail.com ስልክ +251960808193

በጥናቱ ላይ ለመሳተፍ ፈቃደኛ ነዎት? **አመሰግናለሁ!!!**

ሀ) አዎ ለ) አይደለሁም

ተሳታፊው የቃለ ፈቃደኝነቱን መስጠቱን የሚረጋግጥ የጠያቂው ፊርማ

የጠያቂዉ ስም __________ __________ ፊርማ __________ ቀን ----/-----/--------

የሱፐርቫይዘሩ ስም ________ __________ ፊርማ __________ ቀን ----/-----/--------

<u>የተዋቀረ መጠይቅ በአማርኛ</u>

መመሪያ: የሚከተሉትን ጥያቄዎች ከጠየቁ በኋላ ምርጫ ለቀረበላቸው ጥያቄዎች መልሳቸውን

ያክብቡ ምርጫ ላልቀረበላቸው ጥያቄዎች በተሰጠው ክፍት ቦታ ላይ መልሱን ይፃፉ፡፡

<u>ክፍል1. ማህበራዊ፣ ኢኮኖሚያዊና ዲሞግራፊያዊ ጥያቄዎች</u>

ጥያቄዎች	አማራጭ መልስ	ማስታወሻ
እድሜ (አሁን ላይ)	--	
ፆታ	1. ወንድ<br>2. ሴት	
የጋብቻ ሁኔታ	1. ያገባ/ች<br>2. ያላገባ/ች	
ሀይማኖት	1. ኦርቶዶክስ<br>2. ሙስሊም<br>3. ፕሮቴስታንት<br>4. ሌሎች(ይጠቀሱ)----------------	
የትምህረት ደረጃ	1. ማነበብና መፃፍ የማይችል<br>2. ማንበብና መፃፍ ሚችል (መደበኛ ትምህርት ያልትማረ)<br>3. መጀመሪያ ደረጃ (1-8)<br>4. ሁለተኛ ደረጃ(9-12) እና በላይ	
ስራ	1 የመንግስት ሰራተኛ<br>2 የግል ሰራተኛ<br>3 ነጋዴ<br>4 የቤት እመቤት<br>5 ሌላ(ይጠቀስ)-------------	
የቤተሰቡ ወራዊ ገቢ	-----------------------------------	
ስንት ልጅ አለዎት?	--------------------------------	
እድሜቸዉ ከአምስት አመት በታች የሆኑ ስንት ናቸዉ?	--	
ከህፃኑ ጋር ያለዎት ዝምድና	1. እናት ወይም አባት<br>2. አያት<br>3. የስጋ ዘመድ<br>4. ሌሎች-------	

ክፍል ሁለት፡ ጤና ነክ ጥያቄዎች

ጥያቄዎች	መልስ	ማስታወሻ
ልጅዎን ከአሁን በፊት አስከትበዉ ያቃሉ	1. አወ<br>2. አይደለም	
በባለፈዉ አንድ አመት ዉስጥ ዉባ ታመዉ ያቃሉ	1. አወ<br>2. አይደለም	
ልጅዎ በባለፈዉ አንድ አመት ዉስጥ ዉባ ታሞ/ማ ያዉቃል/ታዉቃለች	1. አወ<br>2. አይደለም	

ክፍል ሶስት፡ እዉቀት እና ፈቃደኝነት ነክ ጥያቄዎች

ጥያቄ	መልስ	ማስታወሻ
ስለዉባ ክትባት ሰምተዉ ያቃሉ	1. አወ<br>2. አይደለም	
መልሱ አወ ከሆነ፣ መረጃዉን ከየት አገኙት	1. ከመንግስት የዜና አዉታሮች (ቲቪ፣ሬድዮ)<br>2. ከመንግስት አካላት<br>3. ከማህበራዊ ሚድያ(ፌስቡክ፣ቴሌግራም)<br>4. ከጓደኛ እና ከዘመድ<br>5. ከጤና ባለሙያወች<br>6. ሌሎች-------	
ስለዉባ ክትባት ጥቅም ያቃሉ	1. አወ<br>2. አይደለም	
የወባ ክትባት ለየትኞቹ የእድሜ ክልል ህፃናት ይሰጣል	1. ከአምስት አመት በታች<br>2. ከአምስት አመት በላይ<br>3. አላቅም	
የወባ ክትባትን የመከላከል አቅም ያቃሉ	1. አወ<br>2. አይደለም	
ወባን ለመከላከል ስንት ጊዜ መከተብ አለባቸዉ	1. ሁለት ጊዜ<br>2. አራት ጊዜ<br>3. አላቅም	
የወባ ክትባት የጎንዮሽ ጠንቅ አለዉ	1. አወ<br>2. አይደለም	
ከመጠን ያለፈ የወባ ክትባት መዉሰድ ለጤና ችግር ያመጣል	1. አወ<br>2. አይደለም	
ለወደፊት የወባ ክትባት ሲኖር ህፃኑን/ኗን ለማስከተብ ፈቃደኛ ነዎት	1. አወ<br>2. አይደለም	

ፈቃደኛ ካልሆኑ ፣ለምን	-----------------------------	

አመሰግናለሁ!!!!
